# Echocardiographic Pulmonary to Left Atrial Ratio (ePLAR): A Comparison Study between Ironman Athletes, Age Matched Controls and A General Community Cohort

**DOI:** 10.3390/jcm8101756

**Published:** 2019-10-22

**Authors:** Mai Tran, Agatha Kwon, David Holt, Rebecca Kierle, Benjamin Fitzgerald, Isabel Scalia, William Scalia, Geoffrey Holt, Gregory Scalia

**Affiliations:** 1 Royal Brisbane and Women’s Hospital, Herston 4029, Australia; m.tra92@gmail.com (M.T.); holty_91@hotmail.com (D.H.); igscalia@gmail.com (I.S.); 2 Genesis Care, Auchenflower 4066, Australia; akwon@heartcarepartners.com.au (A.K.); Rebecca.Kierle@genesiscare.com (R.K.); bmcd124@yahoo.com.au (B.F.); GWHolt@heartcarepartners.com.au (G.H.); 3 The Prince Charles Hospital, Brisbane 4032, Australia; will.scalia2@gmail.com; 4 Department of Medicine, University of Queensland, Brisbane 4032, Australia

**Keywords:** exercise physiology, echocardiography, pulmonary and systemic vascular resistance, ironman athletes, ePLAR

## Abstract

Background: During exercise there is a proportionally lower rise in systemic and pulmonary pressures compared to cardiac output due to reduced vascular resistance. Invasive exercise data suggest that systemic vascular resistance reduces more than pulmonary vascular resistance. The aim of this study was the non-invasive assessment of exercise hemodynamics in ironman athletes, compared with an age matched control group and a larger general community cohort. Methods: 20 ironman athletes (40 ± 11 years, 17 male) were compared with 20 age matched non-athlete controls (43 ± 7 years, 10 male) and a general community cohort of 230 non-athletes individuals (66 ± 11 years, 155 male), at rest and after maximal-symptom limited treadmill exercise stress echocardiography. Left heart parameters (mitral E-wave, e’-wave and E/e’) and right heart parameters (tricuspid regurgitation maximum velocity and right ventricular systolic pressure), were used to calculate the echocardiographic Pulmonary to Left Atrial Ratio (ePLAR) value of the three groups. Results: Athletes exercised for 12.2 ± 0.53 min, age matched controls for 10.1 ± 2.8 min and general community cohort for 8.3 ± 2.6 min. Mitral E/e’ rose slightly for athletes (0.9 ± 1.8), age matched controls (0.6 ± 3.0) and non-athletes (0.4 ± 3.2). Right ventricular systolic pressure increased significantly more in athletes than in both non-athlete cohorts (35.6 ± 17 mmHg vs. 20.4 ± 10.8 mmHg and 18 ± 9.6 mmHg). The marker of trans-pulmonary gradient, ePLAR, rose significantly more in athletes than in both non-athlete groups (0.15 ± 0.1 m/s vs. 0.07 ± 0.1 m/s). Conclusions: Pulmonary pressures increased proportionally four-fold compared with systemic pressures in ironman athletes. This increase in pulmonary vascular resistance corresponded with a two-fold increase in ePLAR. These changes were exaggerated compared with both non-ironman cohorts. Such changes have been previously suggested to lead to right ventricle dysfunction, arrhythmias and sudden cardiac death.

## 1. Introduction

Ultra-elite, ironman-level athletes have extreme levels of cardiovascular fitness and adaptation, allowing for very prolonged periods of high aerobic cardiac output [1]. Both left and right ventricles are exposed to these high output states. The relative load on each ventricle with exercise would be dependent on the vascular resistance in the systemic and pulmonary circuits, respectively. Adverse cardiac outcomes in elite athletes have been ascribed to morphologic changes in the right ventricle (disproportionate to the left ventricle). Right heart dilatation and atrial arrhythmias are relatively common in elite athletes [2,3,4,5]. Malignant rhythms have also been reported [2].

The ability to assess right and left heart hemodynamics non-invasively with echocardiography, at rest, is well established. Peak-exercise Doppler assessment of left and right flows is now achievable in most subjects using modern ultrasound equipment. Assessing the changes in volume and pressure parameters from rest to peak exercise in elite athletes, in comparison to non-athlete groups, was undertaken, to highlight adaptive changes in circulatory exercise response.

It is hypothesized that the prolonged maximal cardiac output levels generated by elite ironman-level athletes will be associated with a disproportionate increase of pulmonary pressures compared with systemic pressures, as well as with associated increases in trans-pulmonary gradients (as measured by the echocardiographic Pulmonary to Left Atrial Ratio (ePLAR)), compared with non-athlete, age-matched control and general community subjects.

## 2. Methods

### 2.1. Subject Selection and Exercise Protocols

This study therefore analyzed athletes, who were in training or had completed an ironman-level event (3.8 km swim, 180 km cycle and 42.2 km run), compared with two population samples of well, non-athlete subjects. These two populations were a small group of age matched controls (AMC) and a much larger general community cohort (GCC). The athlete population were volunteer subjects. The non-athlete population consisted of consecutive patients referred for exercise stress echocardiography, for investigation for inducible ischemia or causes of exertional breathlessness. Subjects with positive stress echocardiography were excluded from the study group. Subjects were excluded if they were taking beta-blockers, had arrhythmias or had mitral valve prostheses. Informed consent for stress echocardiography was obtained from each individual included in this study. In addition, this study conforms to the ethical guidelines of the 1975 declaration of Helsinki [6].

Groups underwent treadmill exercise stress echocardiography. Athletes were exercised using a two-minute accelerated Bruce Protocol. Non-athletes were exercised using the standard clinical three minutes Bruce protocol. The difference in protocol was due to the ability of the athletes to accelerate through the protocol, given their fitness level. Groups were exercised until fatigue. Continuous electrocardiographic monitor data and interval manual systemic blood pressures were acquired.

### 2.2. Echocardiographic Methods

Comprehensive non-invasive hemodynamics and structural echocardiographic assessment was undertaken before exercise. No contrast agent or agitate saline was used. Doppler assessment of trans-mitral flow, mitral annular Doppler tissue imaging velocities and tricuspid regurgitation velocity were assessed in relaxed apnea prior to exercise. These measurements were repeated quickly in forced expiration immediately after exercise. The echocardiographic Pulmonary to Left Atrial Ratio (ePLAR) was calculated from resting and peak-exercise data, via the equation for ePLAR [7]:

$$\mathrm{ePLAR}\;\left(m/s\right)=\frac{\mathrm{TR}\;\mathrm{Vmax}\;\left(m/s\right)}{\mathrm{mitral}\;E/e’}$$

Cardiac output was calculated in all subjects from LVOT (left ventricular outflow tract) diameter and velocity time integral, and heart rate at rest and at peak exercise. Systemic vascular resistance (SVR) was calculated in athletes from mean arterial pressure and left heart cardiac output, via the formula [8]:

$$\mathrm{SVR}=80\;\times \;\frac{\mathrm{mean}\;\mathrm{arterial}\;\mathrm{pressure}}{HR\;x\;LVOT\;VTI\;x\;{\left(LVOT\;diameter\right)}^{2}x\;0.785\;}$$

Pulmonary vascular resistance (PVR) was calculated in athletes, via the formula [9]:

$$PVR=0.16+\left(\frac{10\;x\;TR\;V_{max}}{RVOT\;VTI}\;\right)$$

All Doppler measurements were recorded using three beat capture. Numerical measurements were made by an experienced sonographer. All results were then examined and, if necessary, repeat measurements were taken by a sub-specialty trained stress echocardiography physician.

### 2.3. Statistical Methodology

Categorical variables were expressed as absolute values and continuous variables were expressed as mean ± standard deviation (±). Continuous variables were compared by normalized t-tests. “Analyze-it” software was used for statistical analysis. A *p*-value of <0.05 was considered statistically significant. All hemodynamic and echocardiographic data were examined for normal distribution, using Kurtosis and skewness tests, with limits of −2.0 to +2.0 for each considered to be indicative of normal distribution. Multiple linear regression analysis using SPSS Software (IMB Corp) assessed for interaction of variable effects on the primary dependent variable (ΔePLAR).

## 3. Results

### 3.1. Resting Echo Parameters

At rest, echocardiographic findings showed similar left ventricular systolic function in the athletes and non-athletes by ejection fraction (63.7 ± 3.5% vs. AMC 65.9 ± 3.6, *p* = ns vs. GCC 64.8 ± 5.3%, *p* = ns). Left heart Doppler flow parameters showed similar trans-mitral Doppler E-wave velocities in athletes (0.8 ± 0.1 m/s) and non-athletes (AMC 0.87 ± 0.3 m/s, *p* = ns and GCC 0.8 ± 0.2 m/s, *p* = ns) (see Table 1). However, athletes and their AMCs had more vigorous Doppler tissue velocities of the septal mitral annulus at rest, with e’-waves 0.11 ± 0.02 m/s and 0.1 ± 0.02 m/s respectively (*p* = ns), compared with GCC’s e’-wave of 0.08 ± 0.03 m/s (*p* < 0.001). These data yielded lower mitral E/e’ values in athletes (7.6 ± 1.3) than non-athletes (AMC 9.0 ± 4.1, *p* = ns and GCC 9.8 ± 3.8, *p* = 0.01), consistent with lower left atrial filling pressures in the athletes (see Table 1).

Tricuspid regurgitation peak Doppler velocity at rest was similar in athletes (2.4 ± 0.2 m/s) and non-athletes (AMC 2.3 ± 0.2 m/s, *p* = ns and GCC 2.4 ± 0.3 m/s, *p* = ns). Calculated resting right ventricular systolic pressure was also not significantly different in athletes (26.4 ± 3.7 mmHg), compared to non-athletes (AMC 26.6 ± 3.7 mmHg, *p* = ns and GCC 26.9 ± 5.6 mmHg, *p* = ns) (see Table 2). The combination of similar tricuspid velocities with lower trans-mitral E/e’ values in the athletes yielded higher resting ePLAR values (0.33 ± 0.06 m/s,) than non-athletes (AMC 0.30 ± 0.1 m/s, *p* = ns and GCC 0.27 ± 0.09 m/s, *p* < 001) (see Table 2). This is consistent with higher resting trans-pulmonary gradient estimations in athletes than non-athletes.

### 3.2. Exercise Performance

Athletic subjects (*n* = 20, age 39.5 ± 11.3 years, 17 male) exercised for 12.2 ± 0.53 min on the accelerated protocol (completing stage 6 in most cases). They achieved a maximum heart rate rise from 57.5 ± 10 beats per minute to 175.6 ± 10.9 beats per minute (see Table 2). This was associated with an increase in systolic pressure from 120.8 ± 12.9 mmHg to 170.5 ± 12.6 mmHg (see Table 2). AMC (*n*= 20, age 42.6 ± 7.0 years, 10 male) exercised for 10.1 ± 2.8 min on the standard Bruce protocol. GCC (*n*= 230, age 66.0 ± 10.7 years, 155 male), exercised for 8.3 ± 2.6 min on the standard Bruce protocol (on average not quite completing stage 3). Heart rate rose from 69.6 ± 11.1 beats per minute to 169.9 ± 16.8 beats per minute in AMC, and 69.5 ± 10.6 beats per minute to 146 ± 20.6 beats per minute in the GCC (see Table 2). Systolic blood pressure rose from 116.3 ± 14.5 mmHg to 165.6 ± 28.7 mmHg in AMC, and 126.6 ± 18.9 mmHg to 172.5 ± 24.6 mmHg in GCC (see Table 1).

### 3.3. Cardiac Output Response to Exercise

**Figure 1:**
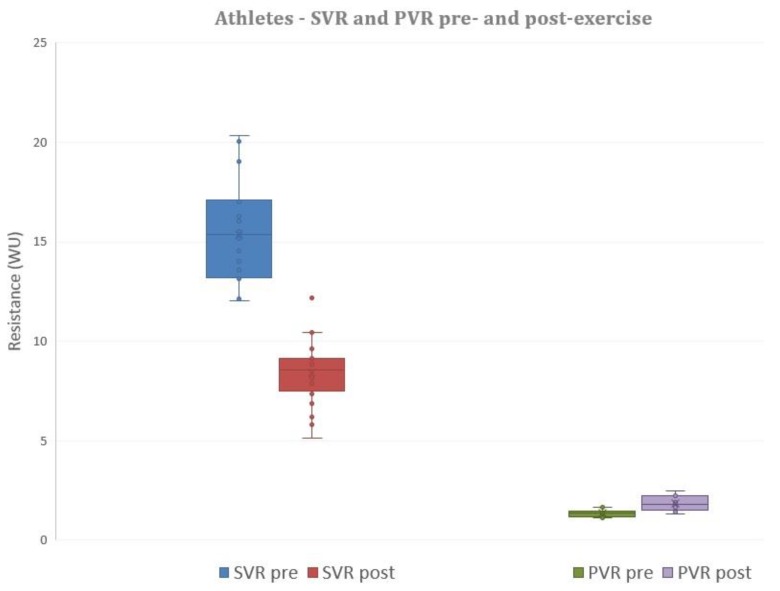
Systemic vascular resistance (SVR) decreases significantly from pre- to post-exercise in the athlete population. Pulmonary vascular resistance (PVR) increases significantly with exercise. The box and whisker plots depict the minimum, first quartile, median, third quartile and maximum values of each data set.

In the athletes, left heart cardiac output rose from 6.0 ± 1.2 L/min to 13.3 ± 2.97 L/min (*p* < 0.001). In the AMC group, cardiac output rose less than the athletes’, from 5.5 ± 1.0 L/min at rest to 10.1 ± 3.6 L/min at peak exercise. In the GCC group, cardiac output rose less than the athletes’, from 5.7 ± 1.4 L/min at rest to 9.2 ± 2.5 L/min at peak exercise. In athletes, right heart cardiac output rose from 6.3 ± 1.5 l/min to 14.9 ± 4.5 L/min (*p* < 0.001). Left and right heart flows were not different before and after exercise (*p* = ns for comparisons). Calculated systemic vascular resistance fell with exercise, from 15.2 ± 2.5 WU to 8.3 ± 1.6 WU (*p* < 0.001). Pulmonary vascular resistance, however, rose from 1.33 ± 0.16 WU to 1.86 ± 0.38 WU (*p* < 0.001) (see Figure 1).

### 3.4. Exercise Hemodynamics

**Figure 2:**
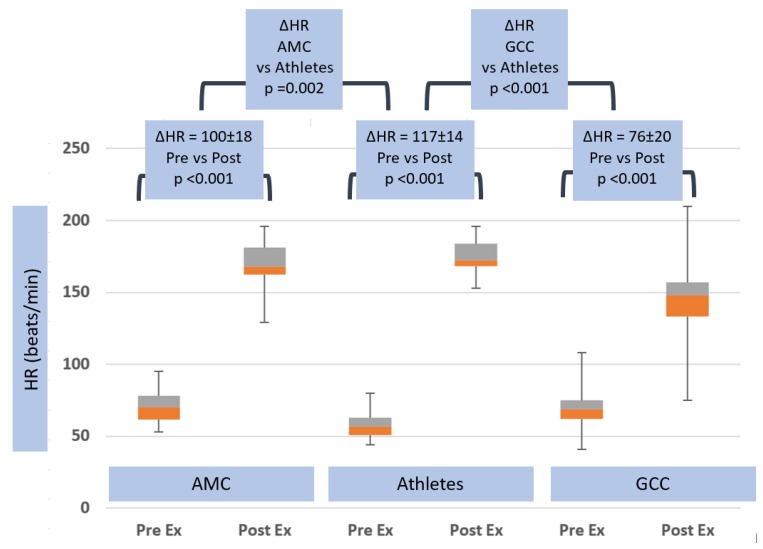
Heart rate response in age matched controls, athletes and general community cohort populations, pre- and post-exercise. The box and whisker plots depict the minimum, first quartile, median, third quartile and maximum values of each data set. The change in heart rate from pre- to post-exercise is shown for the three groups, with standard deviations and *p*-values. The change in heart rate between all groups is also shown, with *p*-value.

**Figure 3:**
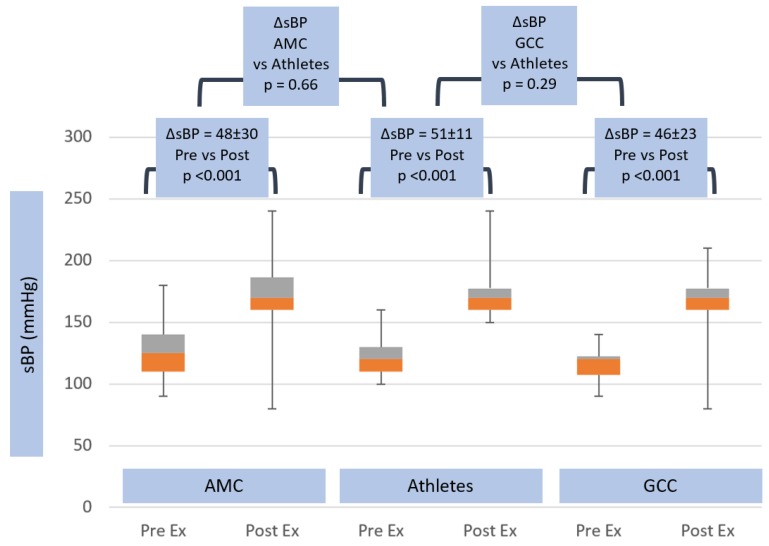
Systolic blood pressure response in age matched controls, athletes and general community cohort populations, pre- and post-exercise. The box and whisker plots depict the minimum, first quartile, median, third quartile and maximum values of each data set. The change in systolic blood pressure from pre- to post-exercise is shown for the three groups, with standard deviations and *p*-values. The change in systolic blood pressure between all groups is also shown, with *p*-value.

There were substantial differences in exercise hemodynamic changes in athletes versus non-athletes. Heart rate increased (ΔHR) 117 ± 14.2 beats per minute in athletes, compared with 100.0 ± 18.2 beats per minute in AMC (*p* = 0.002) and 76 ± 20.1 beats per minute in GCC groups (*p* < 0.001) (see Table 2 and Figure 2). Systolic blood pressure rose, however, to a similar degree in both groups, (ΔsBP) 51.3 ± 11.1 mmHg vs. 48.1 ± 29.7 mmHg in AMC, *p* = ns, and 45.6 ± 23.3 mmHg in GCC, *p* = ns (see Table 2 and Figure 3).

### 3.5. Exercise Echocardiographic Parameters

**Figure 4:**
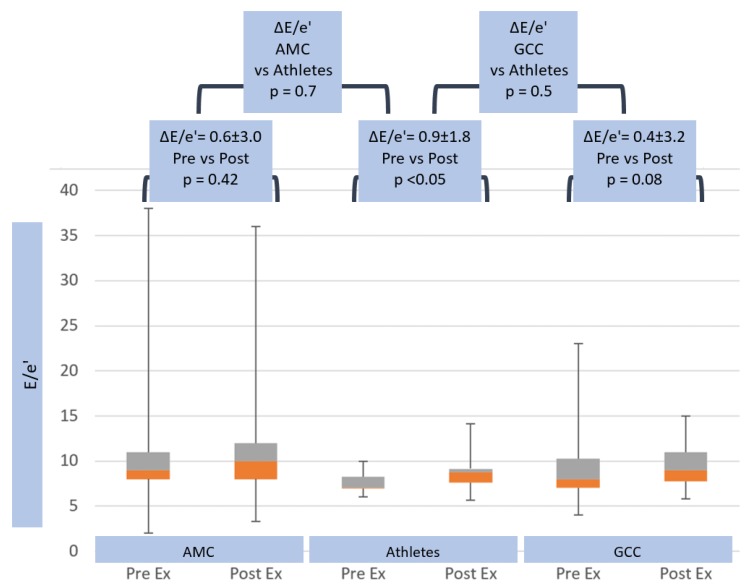
Trans-mitral E/e’ response in age matched controls, athletes and general community cohort populations, pre- and post-exercise. The box and whisker plots depict the minimum, first quartile, median, third quartile and maximum values of each data set. The change in E/e’ from pre- to post-exercise is shown for the three groups, with standard deviations and *p*-values. The change in E/e’ between all groups is also shown, with *p*-value.

At peak exercise, trans-mitral Doppler E-wave rose to 1.3 ± 0.2 m/s in athletes and 1.3 ± 0.3 m/s (*p* = ns) in AMC, which was significantly higher than GCC (1.1 ± 0.3 m/s, *p* < 0.001) (see Table 1). Mitral annular e’-wave increased to similar levels of 0.16 ± 0.03 m/s in athletes and non-athletes (AMC 0.13 ± 0.02, *p* ≤ 0.05 and GCC 0.13 ± 0.11 m/s, *p* = ns) (see Table 1). The left heart filling parameter E/e’ increased to 8.5 ± 2.0 in athletes and in non-athletes (AMC 9.6 ± 2.7, *p* = ns and GCC 10.2 ± 3.7, *p* = 0.04) (see Table 1). Left heart parameters showed significantly greater trans-mitral ΔE-wave velocities in athletes (0.5 ± 0.2 m/s), compared with GCC (0.3 ± 0.2 m/s, *p* = 0.003), but parameters were similar to non-athletes AMC 0.4 ± 0.2, *p* = 0.05 (see Table 2). However, Δe’-wave (0.05 ± 0.02 m/s vs. 0.03 ± 0.02 m/s, *p* = ns in AMC, and 0.04 ± 0.11 m/s, *p* = ns in GCC) and ΔE/e’ (0.9 ± 1.8 vs. 0.6 ± 3.0, *p* = ns in AMC, and 0.4 ± 3.2, *p* = ns in GCC) (see Table 2 and Figure 4) were not significantly different between the groups.

**Figure 5:**
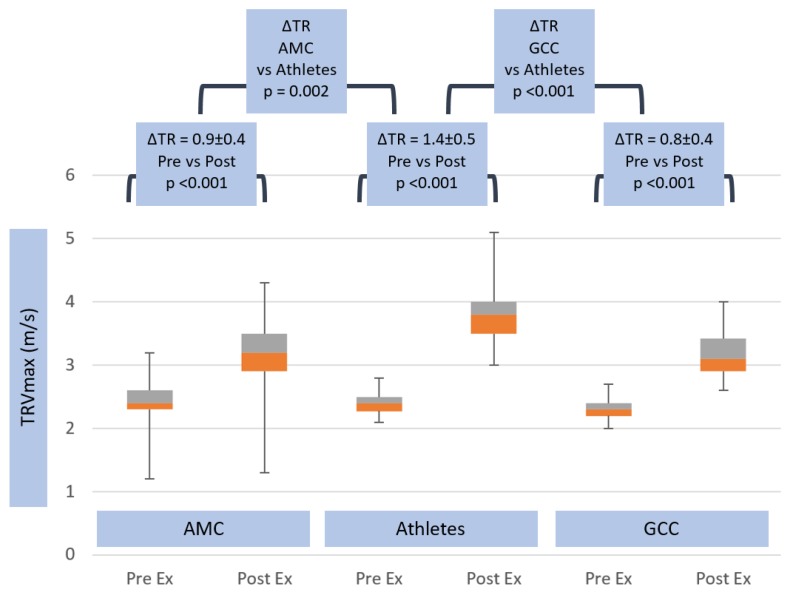
Tricuspid valve TR Vmax response in age matched controls, athletes and general community cohort populations, pre- and post-exercise. The box and whisker plots depict the minimum, first quartile, median, third quartile and maximum values of each data set. The change in TR Vmax from pre- to post-exercise is shown for the three groups, with standard deviations and *p*-values. The change in TR Vmax between all groups is also shown, with *p*-value.

**Figure 6:**
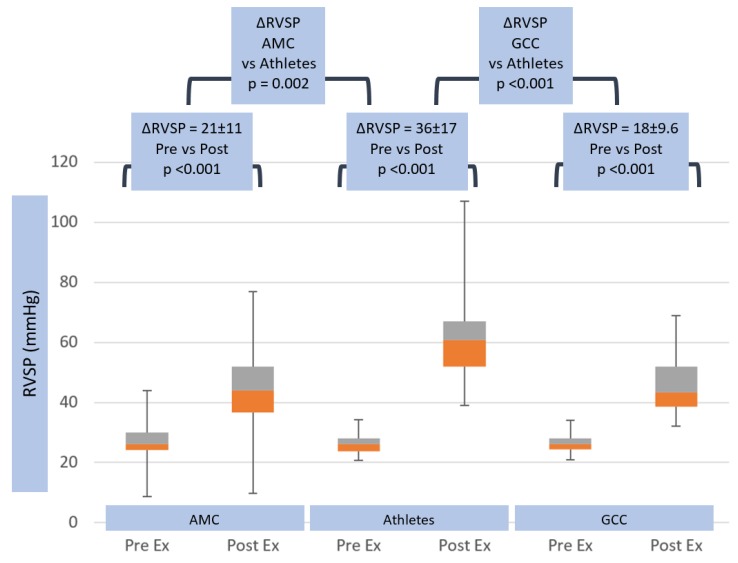
Right ventricular systolic pressure (RVSP) response in age matched controls, athletes and general community cohort populations, pre- and post-exercise. The box and whisker plots depict the minimum, first quartile, median, third quartile and maximum values of each data set. The change in RVSP from pre- to post-exercise is shown for the three groups, with standard deviations and *p*-values. The change in RVSP between all groups is also shown, with *p*-value.

Peak tricuspid valve regurgitation velocity rose to higher values (3.8 ± 0.5 m/s) in athletes than non-athletes (AMC and GCC 3.2 ± 0.4 m/s, *p* < 0.001) (see Table 1). This yielded significantly higher exercise RVSP values of 62 ± 17 mmHg in athletes, compared with 47 ± 11 mmHg (*p* ≤ 0.05) in AMC and 45.1 ± 11.1 mmHg in GCC (*p* < 0.001) (see Table 1). These relative changes in right and left heart parameters at peak exercise generated significantly higher peak-exercise ePLAR values in athletes (0.47 ± 0.13 m/s) than non-athletes (AMC 0.37 ± 0.1 m/s, *p* ≤ 0.05 and GCC 0.35 ± 0.11 m/s, *p* < 0.001) (see Table 1). Tricuspid regurgitation velocity rose significantly more in athletes (ΔTRV_max_ 1.4 ± 0.5 m/s) than in non-athletes (ΔTRV_max_ 0.9 ± 0.4 m/s, *p* = 0.002 in AMC and 0.8 ± 0.4 m/s, *p* < 0.001 in GCC) (see Table 2 and Figure 5). This yielded ΔRVSP changes of 35.6 ± 17 mmHg in athletes, compared with 20.4 ± 10.8 mmHg (*p* = 0.002) in AMC, and 18 ± 9.6 mmHg (*p* < 0.001) in GCC (see Table 2 and Figure 6).

**Figure 7:**
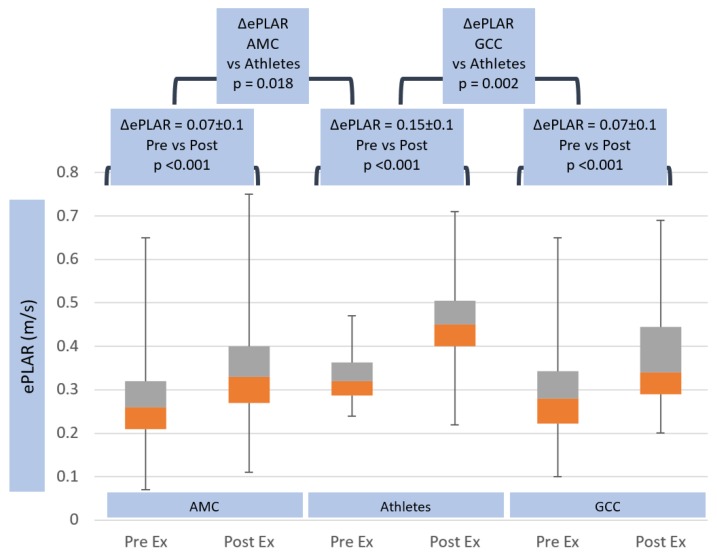
Trans-pulmonary gradient as assessed by ePLAR response in age matched controls, athletes and general community cohort populations, pre- and post-exercise. The box and whisker plots depict the minimum, first quartile, median, third quartile and maximum values of each data set. The change in ePLAR from pre- to post-exercise is shown for the three groups, with standard deviations and *p*-values. The change in ePLAR between all groups is also shown, with *p*-value.

Calculated ePLAR, as a marker of trans-pulmonary gradient, rose significantly more in athletes (ΔePLAR 0.15 ± 0.1 m/s) compared with both non-athlete groups (ΔePLAR 0.07 ± 0.1 m/s, *p* < 0.05) (see Table 2 and Figure 7). Multiple linear regression analysis was tested for the interaction of athlete status, METS, age, BSA, ΔSPB, HR_max_ and ΔCO. Athlete status was the most powerful predictor of ΔePLAR, with a point estimate of 39% increase in ΔePLAR for athlete status (range 1.9–76.7, *p* = 0.04, see Table 3). Interestingly, METS significantly contributed to the prediction of ΔePLAR, but increasing METS by 1 unit actually lowered ΔePLAR by 3.9% (range −7.3 to −0.5, *p* = 0.025). None of the other tested parameters significantly influenced ΔePLAR.

## 4. Discussion

Some endurance athletes are vulnerable to cardiovascular complications, such as ventricular arrhythmias, due to right ventricular dysfunction [2,3,4,5]. This dysfunction has been proposed as a possible aetiology of sudden cardiac death [2,3,4,5], with an incidence of 0.6–3.6 per 100,000 athletes [10]. A potential mechanism for this increased risk is a progressive and disproportionate increase in pulmonary arterial pressures with exercise intensity, which leads to a greater afterload on the right ventricle [11,12]. With intense exercise, this hemodynamic right heart load results in excess myocardial work and wall stress [13], which promotes structural, functional and arrhythmic remodeling of the right ventricle [13,14,15,16,17,18,19].

Elite athletes sustain cardiac outputs up to three times baseline for many hours at a time [1]. Our athletes increased their cardiac output by just over double the baseline. This achieved an increased heart rate, stroke volume and decreased systemic vascular resistance (SVR) [11], to allow for increased blood flow to exercising muscles. Systemic vascular resistance (SVR) decreased with exercise, significantly more than pulmonary vascular resistance (PVR). Our athletes decreased SVR by 45% (see Figure 1). This decrease in systemic vascular resistance is reflected by a relatively modest rise in mean systemic blood pressure (+50% in our athletes) with exertion compared to cardiac output [20] (+100%–130% in this study). The ability to decrease systemic vascular resistance is improved by exercise training [21] via enhanced release of endogenous nitric oxide, resulting in systemic vasodilation [22]. The decrease in systemic vascular resistance is important, as systemic pressures during exercise are required to equilibrate at modestly increased levels [11]. There are prognostic implications involved—the greater the elevation in systemic pressures, the greater the association with an increased risk of stroke [23].

Pulmonary pressures also rise with the increased cardiac output of aerobic exercise [24,25]. The pulmonary arterial vascular bed is a lower-pressure system. This equates to a lower-resistance system [24] with an extensive branching of vessels, a more compliant vasculature due to thin walls, and hypoxia-induced vasoconstriction [24]. As the pulmonary arterial system is already low resistance, there is limited capacity for further decreases in PVR with exertion [26]. Mechanisms in which pulmonary vascular resistance decreases during exercise include distension of the circulatory system volume, vasodilation—mainly mediated by nitric oxide—and an increase in cardiac output of five times the normal baseline [27]. With these mechanisms, the reduction in PVR has been reported at 20%–50% [28], which is limited when compared to the profound reductions in systemic vascular resistance. The ability of the pulmonary vasculature to accommodate these prolonged high-flow rates remains to be quantified in ultra-elite athletes [24]. However, in our elite athletes, PVR rose by 40% with exercise (see Figure 1).

Much of the foundational understanding of exercise changes in the circulation come from invasive hemodynamics (peripheral arterial and pulmonary arterial) in the cardiac catheter laboratory, using supine cycle ergometers on the table. These sophisticated but invasive studies have shown a strong linear relationship between increase in heart rate and mean pulmonary arterial pressure [29]. In patients with heart failure, with reduced ejection fraction and post-capillary pulmonary hypertension, lowering filling pressures improved exercise pulmonary hemodynamics. Disproportionate increases in pulmonary pressures and trans-pulmonary gradient are predictors of pre-capillary causes of progressive pulmonary hypertension [30].

Comprehensive Doppler echocardiography is well validated and clinically ubiquitous in its use for assessment of left heart pressures (trans-aortic and left atrial filling pressures) and right heart parameters (right ventricular systolic pressure and pulmonary vascular resistance). Derived systemic vascular resistance can be calculated from non-invasive systolic blood pressure and echocardiographic derived cardiac output. Recently, the trans-pulmonary gradient has been approximated by the newly derived parameter echocardiographic Pulmonary to Left Atrial Ratio (ePLAR), calculated from the maximum tricuspid regurgitation continuous wave Doppler velocity and the trans-mitral E-wave: mitral annular DTI e’-wave (ePLAR (m/s) = TR V_max_/E/e’)) [7].

The availability and ease of administration of these non-invasive assessments of exercise hemodynamics allows the application of such an assessment to more broad community samples to compare with the athlete subgroup. Adaptive differences in physiology become apparent. As expected, athletes achieved higher rate–pressure products at a higher workload than non-athletes. They have similar resting right heart systolic pressures (as assessed by TR V_max_), but lower left atrial filling pressure (as indicated by trans-mitral E/e’). Because of these parameters, trans-pulmonary gradient (as indicated by ePLAR) was higher at rest in the athletes.

The changes in non-invasive hemodynamic parameters with exercise were illuminating. Whilst athletes achieved a higher maximum heart rate than non-athletes, the peak systolic arterial pressure and the ∆SBP with exercise were not significantly different between the three groups. In contrast, however, whilst resting right heart pressures were similar in the three groups, the athletes had a dramatically greater increase in TR V_max_ than non-athletes (+1.4 ± 0.5 m/s vs. AMC +0.9 ± 0.4 mmHg and GCC +0.8 ± 0.4 m/s) and RVSP (+36 ± 17 mmHg vs. AMC +20.4 ± 10.8 mmHg and GCC +18 ± 10 mmHg). Bossone et al. [31] had similar results, reporting an athlete population with higher TR V_max_ than controls during exercise. The study attributed this phenomenon to higher SV and CO in athletes.

Left heart filling pressure, as indicated by trans-mitral E/e’, was persistently lower in athletes than non-athletes at peak exercise. There was no difference in the change in E/e’ with exercise in the three groups. Because of the substantial increase in right heart systolic pressures (as indicated by TR V_max_), trans-pulmonary gradient (as indicated by ePLAR) rose substantially more in athletes than non-athletes (+0.15 ± 0.1 m/s vs. +0.07 ± 0.1 m/s in both AMC and GCC groups). This supports the relative inability of the pulmonary vascular tree to dilate sufficiently to these very high cardiac output states. La Gerche et al. [13] also found this, in their study of 39 endurance athletes vs. 14 non-athletes via cardiac MRI, which showed greater RV enlargement and wall thickening in the former, given the disproportionate RV load excess.

The greater increase in ePLAR values in athletes, compared to a general community cohort, was expected, since a lower ePLAR value suggests left ventricular diastolic dysfunction, with elevated left heart filling pressures. The GCC, which consisted of older and less fit individuals, was more likely to have this issue, compared to highly trained individuals, who have well adapted hearts. The starting point of ePLAR for the GCC was lower than that of the athlete population, due to the fact that lower ePLAR values are expected with increased age. The mean age of the GCC was 66 ± 10.7 years, compared to the athlete population of 40 ± 11.3 years. However, there was also a greater increase in ePLAR value in athletes, compared to the age matched controls (43 ± 7.0 years) of a similar age, *p* ≤ 0.05.

Pulmonary vascular resistance and trans-pulmonary gradient are important markers of right heart load. Pulmonary vascular disease [32,33,34] and parenchymal lung disease (e.g., COPD [35,36]), have anatomic and functional flow limitation, causing clinical right heart dysfunction and failure in some patients [37]. In disease states, this is due to vascular bed destruction, with a reduced cross-sectional surface area and compliance [38]. This represents a further mechanism for exercise capacity reduction in conditions such as pulmonary fibrosis and COPD, as there is an inability to adapt to the stresses of exercise, compared with healthy counterparts [39].

An increase in pulmonary vascular resistance may result in complications in the athlete population. These complications include atrial fibrillation and right ventricle (RV) systolic dysfunction [2]. Exercise is generally considered to be a *volume-loaded* dynamic. In ultra-high workload athletes, these data show that the right ventricle is exposed to prolonged pressure-loading, with the potential for hypertrophy and fibrotic changes in the long term. These morphologic RV changes may be the reason for adverse long-term events, such as the increased risk of sudden cardiac death. In agreeance with this, La Gerche et al. [2] have suggested echocardiogram and cardiac MRI during exercise, to identify athletes most at risk of RV remodeling and, therefore, RV dysfunction. Heiduchel et al. [3] suggested electrophysiological studies instead, to identify arrhythmia inducibility and mechanism, when confronted with this finding.

## 5. Limitations

One of the limitations of this study is the lack of tricuspid regurgitation velocities available for some patients. TR velocity at peak exercise was only available in 75% available of cases. When the TR velocity was missing, the ePLAR value could not be calculated. Therefore, they were not included in the non-athlete population, as had initially been intended. A lack of cardiac output data in the non-athletes limited the ability to calculate SVR and PVR in the comparative group.

## 6. Conclusions

Systemic vascular resistance decreases with exercise, allowing for prolonged periods of maximal cardiac output with modest elevations in systemic blood pressure. However, in this study of ironman athletes, ePLAR, as a marker of transpulmonary gradient, rose significantly. These changes were exaggerated in comparison to a non-athlete population, which included a group of age matched controls. This hemodynamic right ventricular burden may represent a potential mechanism for increased cardiac risk in the elite athlete population, and of adverse outcomes, such as dangerous arrhythmias and right ventricular dysfunction. This study highlights the value of non-invasive hemodynamic assessment of the right heart and the application of new parameters, such as ePLAR, in delineating pulmonary flow dynamics.

## Figures and Tables

**Table 1:** Mean data and corresponding *p*-values of 20 age matched controls vs. 20 athlete population vs. 230 non-athlete population undergoing treadmill exercise stress echocardiography.

	Age Matched Controls (AMC), *n* = 20	*p*-value, Athletes vs. AMC	Athletes, *n* = 20	*p*-value, Athletes vs. GCC	General Community Cohort (GCC), *n* = 230
Age (years)	42.6 ± 7.0	ns	39.5 ± 11.3	<0.001	66.0 ± 10.7
Sex	10 males		17 males		155 males
BSA (m^2^)	2.0 ± 0.29	ns	2.0 ± 0.15	ns	2.0 ± 0.3
BMI (kg/m^2^)	27.7 ± 7.2	0.01	23.3 ± 2.1	ns	27.4 ± 4.9
Exercise, duration (mins)	10.1 ± 2.8, (Bruce protocol)	<0.05	12.2 ± 0.53 (Athlete protocol)	<0.001	8.3 ± 2.6, (Bruce protocol)
METS, (3.5 mL/min/kg)	10.8 ± 2.6	<0.001	20.4 ± 1.92	<0.001	9.3 ± 2.5
HR pre (beats/min)	69.6 ± 11.1	0.001	57.5 ± 10.0	<0.001	69.5 ± 10.6
sBP pre +(mmHg)	116.3 ± 14.5	ns	120.8 ± 12.9	ns	126.6 ± 18.9
dBP pre (mmHg)	76.5 ± 9.6	ns	72.9 ± 7.7	ns	75.0 ± 9.2
HR max (beats/min)	169.6 ± 16.8	ns	175.6 ± 10.9	<0.001	146.0 ± 20.6
sBP post (mmHg)	165.6 ± 28.7	ns	170.5 ± 12.6	ns	172.5 ± 24.6
dBP post (mmHg)	85.9 ± 20.1	<0.05	74.5 ± 7.2	ns	79.6 ± 15.5
CO (rest), l/min	5.5 ± 1.0	ns	6.0 ± 1.2	ns	5.7 ± 1.4
CO (peak), l/min	10.1 ± 3.6	<0.01	13.3 ± 2.97	<0.001	9.2 ± 2.5
EF pre (%)	65.9 ± 3.6	ns	63.7 ± 3.5	ns	64.8 ± 5.3
TR V_max_ pre (m/s)	2.3 ± 0.2	ns	2.4 ± 0.2	ns	2.4 ± 0.3
RVSP pre (mmHg)	26.6 ± 3.7	ns	26.4 ± 3.7	ns	26.9 ± 5.5
E pre (m/s)	0.87 ± 0.3	ns	0.8 ± 0.1	ns	0.8 ± 0.2
e’ pre (m/s)	0.1 ± 0.02	ns	0.11 ± 0.02	<0.001	0.08 ± 0.03
E/e^’^ pre	9.0 ± 4.1	ns	7.6 ± 1.3	0.01	9.8 ± 3.8
ePLAR pre (m/s)	0.30 ± 0.1	ns	0.33 ± 0.06	<0.001	0.27 ± 0.09
TR V_max_ post (m/s)	3.2 ± 0.4	0.001	3.8 ± 0.5	<0.001	3.2 ± 0.4
RVSP post (mmHg)	47.0 ± 11.0	<0.05	62.0 ± 17	<0.001	45.1 ± 11.1
E post (m/s)	1.3 ± 0.3	ns	1.3 ± 0.2	<0.001	1.1 ± 0.3
e’ post (m/s)	0.13 ± 0.02	<0.05	0.16 ± 0.03	ns	0.13 ± 0.11
E/e^’^ post	9.6 ± 2.7	ns	8.5 ± 2.0	0.04	10.2 ± 3.7
ePLAR post (m/s)	0.37 ± 0.1	<0.05	0.47 ± 0.13	<0.001	0.35 ± 0.11

AMC: age matched controls, GCC: general community cohort, ns: non-significant, BSA: body surface area, BMI: body mass index, METS: metabolic equivalents, HR: heart rate, CO: cardiac output, EF: ejection fraction, TR Vmax: maximum tricuspid regurgitation velocity, RVSP: right ventricular systolic pressure.

**Table 2:** The mean change and corresponding *p*-values in age matched controls vs. athlete population vs. general community cohort from pre- to post-exercise.

	Age Matched Control	*p*-Value Athletes vs. Age Matched Control	Athletes	*p*-Value Athletes vs. on-Athlete Cohort	General Community Cohort
ΔHR (beats/min)	100.0 ± 18.2	0.002	117.1 ± 14.2	<0.001	76.0 ± 20.1
ΔsBP (mmHg)	48.1 ± 29.7	ns	51.3 ± 11.1	ns	45.6 ± 23.3
ΔdBP (mmHg)	8.1 ± 23.9	ns	1.5 ± 4.6	ns	2.9 ± 15.6
ΔTR V_max_ (m/s)	0.9 ± 0.4	0.002	1.4 ± 0.5	<0.001	0.8 ± 0.4
ΔRVSP (mmHg)	20.4 ± 10.8	0.002	35.6 ± 17	<0.001	18 ± 9.6
ΔE (m/s)	0.4 ± 0.2	0.05	0.5 ± 0.2	0.003	0.3 ± 0.2
Δe’ (m/s)	0.03 ± 0.02	ns	0.05 ± 0.02	ns	0.04 ± 0.11
ΔE/e’	0.6 ± 3.0	ns	0.9 ± 1.8	ns	0.4 ± 3.2
ΔePLAR (m/s)	0.07 ± 0.1	0.018	0.15 ± 0.1	0.002	0.07 ± 0.1

HR heart rate, sBP systolic blood pressure, dBP diastolic blood pressure, TR Vmax, maximum tricuspid regurgitation velocity, RVSP right ventricular systolic pressure.

**Table 3:** Multiple linear regression results for predicting ΔePLAR from athlete status, METS (metabolic equivalents), age, BSA (body surface area), ΔsBP (systolic blood pressure), HR_max_ (maximum heart rate) and ΔCO (cardiac output). Only athlete status and METS (negative effect) significantly influenced ΔePLAR.

	Coefficients		Sig.	95.0% Confidence Interval for B	
	Beta	Std. Error		Lower Bound	Upper Bound
Athlete	39.310	18.913	0.040	1.903	76.717
METs	−3.908	1.719	0.025	−7.308	−0.508
Delta CO %	0.019	0.270	0.945	−0.516	0.553
Delta sBP %	0.373	0.212	0.081	−0.047	0.793
Age	−0.510	0.408	0.214	−1.317	0.298
BSA	2.238	14.826	0.880	−27.085	31.561
HR max	−0.001	0.216	0.996	−0.428	0.426
Constant	82.690	61.728	0.183	−39.397	204.777

METS: metabolic equivalents, BSA: body surface area, ΔsBP: systolic blood pressure, HR_max_: maximum heart rate, ΔCO: cardiac output.

## Abbreviations

BSA	Body surface area
BMI	Body mass index
HR	Heart rate
SV	Stroke volume
CO	Cardiac output
sBP	Systolic blood pressure
METS	Metabolic equivalents
dBP	Diastolic blood pressure
MAP	Mean arterial pressure
SVR	Systemic vascular resistance
PVR	Pulmonary vascular resistance
EF	Ejection fraction
E	Trans-mitral Doppler E-wave velocity
e’	Mitral annular e’ wave velocity
E/e’	Trans-mitral E-wave: mitral annular e’ wave
ePLAR	echocardiographic Pulmonary to Left Atrial Ratio
TR V_max_	Maximum tricuspid regurgitation velocity
RVSP	Right ventricular systolic pressure
RVOT	Right ventricular outflow tract
LVOT	Left ventricular outflow tract
VTI	Velocity time integral
